# Shear bond strength of metallic brackets bonded to enamel pretreated with CPP-ACP: a systematic review and meta-analysis of in vitro studies

**DOI:** 10.1186/s12903-023-03103-x

**Published:** 2023-07-01

**Authors:** Yomna M. Yacout, Yomna A. Nabawy, Nadia M. El-Harouni, Tarek N. Yousry

**Affiliations:** 1 Department of Orthodontics, Faculty of Dentistry, Alexandria University, Champollion St, Azarita, P. O. Box: 21521, Alexandria, Egypt; 2 Department of Orthodontics, College of Dentistry El Alamein, Arab Academy for Science, Technology and Maritime Transport (AASTMT), El Alamein, Egypt; 3 Department of Orthodontics, Faculty of Dentistry, Alexandria University, Alexandria, Egypt

**Keywords:** Shear bond strength, Casein phosphopeptide-amorphous calcium phosphate, CPP-ACP, Orthodontic brackets

## Abstract

**Background:**

Development of white spot lesions (WSLs) is common among orthodontic patients. Several measures have been introduced to prevent and remineralize the lesions. Casein phosphopeptide-amorphous calcium phosphate (CPP-ACP) is used for both prevention and remineralization. The effect of its application before bonding is controversial. This systematic review was conducted to investigate the most up to date available literature regarding the effect of CPP-ACP enamel pre-treatment on shear bond strength (SBS) of metallic orthodontic brackets.

**Methods:**

A search was conducted in electronic databases (MEDLINE (via PubMed), Scopus, Cochrane Library, Web of Science and Google scholar (grey literature)) up to March 29^th^, 2023. The inclusion criteria included in vitro studies comparing the SBS of metal orthodontic brackets following pre-treatment of enamel using CPP-ACP versus control. The exclusion criteria included study types other than in vitro studies, studies conducted on non-human enamel, or studies using CPP-ACP in combination with another intervention. The included studies were analysed by two reviewers, independently. The risk of bias assessment was done using a modified risk of bias tool. A Meta-analysis was performed. I^2^ values and Q-test were used for assessment of heterogeneity. Results were displayed in forest plots with a random-effects model. Standardized mean difference, standard error (SE) and 95% confidence intervals were calculated for all studies.

**Results:**

The search resulted in 76 articles. After duplicate removal and assessment for eligibility, 15 studies were included in the review. High statistical heterogeneity was found among the included studies using I^2^ values and Q-Test (I^2^ = 95.147%; Q = 288.456; df = 14; *P* < 0.001). The overall effect of CPP-ACP pre-treatment on the SBS of metal orthodontic brackets was not significant (Mean difference = 1.163 MPa, SE = 0.757, 95% CI = -0.321, 2.648, *p* value = 0.125). The use of CPP-ACP for prevention of WSLs did not significantly affect the SBS of brackets (Standardized mean difference = 1.009, SE = 0.884, 95% CI = -0.723, 2.740, *p* value = 0.254). No significant change was found when CPP-ACP was used for remineralization of WSLs (Standardized mean difference = 1.501, SE = 1.087, 95% CI = -0.630, 3.632, *p* value = 0.167).

**Conclusions:**

Within the limitations of the study, the evidence suggests that the use of CPP-ACP for either prevention or remineralization of WSLs before bonding does not affect the SBS of metal orthodontic brackets.

**Supplementary Information:**

The online version contains supplementary material available at 10.1186/s12903-023-03103-x.

## Background

White spot lesions (WSLs) are a common risk during and after orthodontic treatment especially, in poor oral hygiene patients [1, 2]. These milky white opacities may appear around orthodontic brackets within only 4 weeks of starting treatment [3]. The presence of fixed orthodontic brackets and auxiliaries hampers the maintenance of good oral hygiene, thus resulting in increased food accumulation which increases the risk of WSLs development [2, 4]. In addition, the acid-etching procedure required for bonding orthodontic attachments removes 10–20 μm of the enamel surface, which may increase the risk of enamel demineralization [5]. Furthermore, the increase in the levels of acidogenic bacteria, such as *Streptococcus mutans* and *lactobacilli*, in orthodontic patients after placement of fixed orthodontic appliances lowers the pH of the oral cavity thus favoring enamel demineralization [6, 7]. WSLs may progress into cavitation thus affecting aesthetics and reducing patient satisfaction with the final orthodontic treatment results [8]. Prevention of WSLs starts by educating and motivating the patient to maintain good oral hygiene and consume non cariogenic diet [9]. However, additional preventive measures are often needed to reduce the risk of enamel demineralization in high-risk patients, thus reducing the risk of WSLs formation [10]. One of these measures is the use of casein phosphopeptide-amorphous calcium phosphate (CPP-ACP) [10].

CPP-ACP is a milk-derived bioactive peptide that is available in different forms such as topical dental cream [11], mouth rinse [12], chewing gum [12, 13], and lozenges [14], and it has shown an efficient preventive and remineralizing potential. The suggested anticariogenic mechanism of the CPP-ACP is that it can stabilize calcium and phosphate and preserve them in a soluble form, called amorphous calcium phosphate, providing a reservoir [15]. CPP-ACP can also bind to enamel surface, dental pellicle and dental plaque, thus maintaining a state of calcium and phosphate supersaturation in a close proximity to the tooth structure and a pH buffering action in dental plaque, hence decreasing enamel demineralization and enhancing remineralization [16].

The preservation of sound enamel surface, on the one hand, is important during and after orthodontic treatment. On the other hand, the preventive measures used should not negatively affect the bond strength of the orthodontic brackets. Ideally, the orthodontic bracket shear bond strength (SBS) should range between 5.9 and 7.8 Megapascals (MPa) [17] to withstand the orthodontic and masticatory forces without failure throughout the treatment period and to allow debonding at the conclusion of the treatment without causing enamel damage [18]. Multiple studies have been conducted to evaluate the effects of CPP-ACP pre-treatment on the SBS of orthodontic brackets, however, the results of these studies were controversial. Systematically reviewing the published literature and statistically pooling the data obtained from previous research allows analysis of a larger sample, thus allows the clinician to make evidence-based decisions [19].

Hence, the aim of this systematic review and meta-analysis was to investigate the most up to date available literature regarding the effect of CPP-ACP enamel pre-treatment on the SBS of metallic orthodontic brackets. The review aims to answer the question whether applying CPP-ACP on the enamel for the prevention or treatment of WSLs before bonding affects the SBS of metallic orthodontic brackets.

## Methods

The review and analysis were conducted and reported following the Preferred Reporting Items for Systematic Reviews and Meta-Analyses (PRISMA) statement [20].

### Eligibility criteria

The inclusion criteria were experimental studies conducted on extracted permanent human teeth. The characteristics of the included studies based on PICO [21] were:

- Population (P): Enamel of extracted permanent human teeth.
- Intervention (I): Enamel treatment with CPP-ACP before bonding metallic orthodontic brackets.
- Comparison (C): No enamel pretreatment before bonding metallic orthodontic brackets or treatment with another material.
- Outcome (O): Shear bond strength.

The exclusion criteria included case reports, letters to editor, commentaries, editorials, animal studies, in vivo studies, literature reviews, systematic reviews, and meta-analyses. In addition, studies conducted on non-human enamel, or studies that used CPP-ACP in combination with another intervention were excluded.

### Information sources and search strategy

The detailed search strategy shown in Table 1, was developed with no language, country or publication date restrictions. Five different electronic databases were screened: MEDLINE (via PubMed), Scopus, Cochrane library, Web of Science and Google scholar (Gray literature). To find research that may have been overlooked in the electronic database search, the reference lists of relevant papers were hand-searched. In addition, "Citation Networks" of relevant papers in Web of Science database were checked. Two independent reviewers (YN and YY) searched the literature to find the relevant published studies from the inception of each database up to March 29^th^, 2023.

**Table 1:** Literature search conducted to identify studies. (Last search date March 29^th^, 2023)

Database	Search	Search strategy	Hits
MEDLINE (via PubMed)	#1	"shear strength"[MeSH Terms] OR "shear strength"[Title/Abstract] OR "bond strength"[Title/Abstract] OR "shear bond strength"[Title/Abstract]	21,679
	#2	caseins[MeSH Terms] OR "casein phosphopeptide amorphous calcium phosphate nanocomplex"[Supplementary Concept] OR "casein phosphopeptide amorphous calcium phosphate"[Title/Abstract] OR "cpp acp"[Title/Abstract]	17,173
	#3	"orthodontic brackets"[MeSH Terms] OR "orthodontic bracket*"[Title/Abstract] OR "orthodontic brace*"[Title/Abstract] OR "metal bracket*"[Title/Abstract] OR "metal brace*"[Title/Abstract] OR "metallic bracket*"[Title/Abstract]	5,521
	#4	#1 AND #2 AND #3	**24**
Scopus	#1	TITLE-ABS-KEY (“shear strength” OR “shear bond strength” OR “bond strength”)	130,011
	#2	TITLE-ABS-KEY (“casein phosphopeptide-amorphous calcium phosphate” OR “CPP-ACP”)	820
	#3	TITLE-ABS-KEY ("orthodontic brackets" OR “orthodontic braces” OR “metal* bracket*” OR “metal* brace*”)	6,117
	#4	#1 AND #2 AND #3	**22**
Cochrane	#1	[mh "Shear Strength"] OR “shear strength”:ti,ab,kw OR “bond strength”:ti,ab,kw OR “shear bond strength”:ti,ab,kw	1,149
	#2	[mh "caseins"] OR “casein phosphopeptide-amorphous calcium phosphate”:ti,ab,kw OR “CPP-ACP”:ti,ab,kw	623
	#3	[mh "Orthodontic Brackets"] OR orthodontic NEXT bracket*:ti,ab,kw OR orthodontic NEXT brace*:ti,ab,kw OR metal* NEXT bracket*:ti,ab,kw OR metal* NEXT brace*:ti,ab,kw	981
	#4	#1 AND #2 AND #3	**1**
Web Of Science	#1	(((((TI=("shear strength")) OR TI=("shear bond strength")) OR AB=("shear strength")) OR AB=("shear bond strength")) OR AK=("shear strength")) OR AK=("shear bond strength")	47,946
	#2	(((((TI=("casein phosphopeptide-amorphous calcium phosphate")) OR TI=("CPP-ACP")) OR AB=("casein phosphopeptide-amorphous calcium phosphate ")) OR AB=("CPP-ACP")) OR AK=("casein phosphopeptide-amorphous calcium phosphate")) OR AK=("CPP-ACP")	549
	#3	(((((((((((TI=("orthodontic bracket*")) OR TI=("orthodontic brace*")) OR AB=(" orthodontic bracket*")) OR AB=("orthodontic brace*")) OR AK=(" orthodontic bracket*”)) OR AK=("orthodontic brace*")) OR TI=("metal* bracket*")) OR TI=("metal* brace*")) OR AB=("metal* bracket*")) OR AB=("metal* brace*")) OR AK=("metal* bracket*")) OR AK=("metal* brace*")	2,433
	#4	#1 AND #2 AND #3	**22**
Google scholar https://scholar.google.com.eg/		allintitle: ("shear strength" OR "shear bond strength") AND ("casein phosphopeptide-amorphous calcium phosphate" OR "CPP-ACP") AND ("orthodontic bracket" OR "metal bracket" OR "orthodontic brackets" OR "metal brackets")	**7**

### Study selection

The relevant articles were imported into EndNote X9™ reference manager (Clarivate™, Philadelphia, PA). Duplicates were removed using EndNote’s “Find Duplicates” function and any missed duplicates were removed manually. The titles and abstracts of the articles were then reviewed by two authors (YN and YY) independently to exclude any article that does not follow the inclusion criteria. The full text of potentially eligible articles was assessed for eligibility by the same two reviewers. Any disagreement between the two reviewers was solved by discussion. If the disagreement regarding the eligibility of the studies persisted, a third reviewer opinion (NE) was obtained.

### Data extraction

The data were collected from eligible articles by one author (YN) and revised by another (YY). A data extraction form was created using Microsoft 365® Excel® software (Microsoft Corporation, Redmond, WA). The form included the following information: authors’ names, publication year, total sample size, number of groups, number of samples per group, condition of enamel before bonding, protocol of CPP-ACP application, duration of CPP-ACP application, number of CPP-ACP applications, protocol implemented in control groups, and mean and standard deviation (SD) of SBS in MPa. If any relevant data was missing from a paper, the corresponding author of said paper was contacted by e-mail. If no response was obtained within 2 weeks, another e-mail was sent.

### Risk of bias assessment

Two reviewers (YN and TY) performed the risk of bias assessment independently using a modification of the Risk of Bias tool suggested by Sarkis-Onofre et al. [22]. The risk of bias was assessed based on the description of the following parameters in the article: 1- description of sample-size calculation, 2- randomization of teeth, 3- presence of a control group, 4- using teeth free of caries or restorations, 5- description of sample preparation (handling, cleaning and storage of the teeth), 6- using the materials according to the manufacturer’s instructions, 7- blinding of the outcome assessor, 8- bonding procedure executed by a one investigator. If the parameter was reported by the authors, the specific parameter was marked as “Yes”. If it was not reported or no information could be found, it was marked as “No”. Articles reporting three or less parameters were considered to have a high risk of bias, four or five parameters a medium risk of bias, and six or more parameters a low risk of bias. If no consensus regarding the risk of bias of any article could be reached between the two reviewers (YN and TY), a third reviewer (NE) was consulted.

### Synthesis of results

Meta-analysis was performed using OpenMeta[Analyst] software [23]. For assessment of heterogeneity of the studies I^2^ values and Q-Test were used. The I^2^ Index measures the percentage of variation across studies and represents the heterogeneity (25% corresponds to low heterogeneity, 50% to moderate heterogeneity, and 75% to high heterogeneity). Forest plots with a random-effects model were used in the current study due to the high heterogeneity found among the studies. Standardized mean difference, standard error (SE) and 95% confidence interval (CI) were calculated for all studies.

## Results

### Study selection

**Fig. 1:**
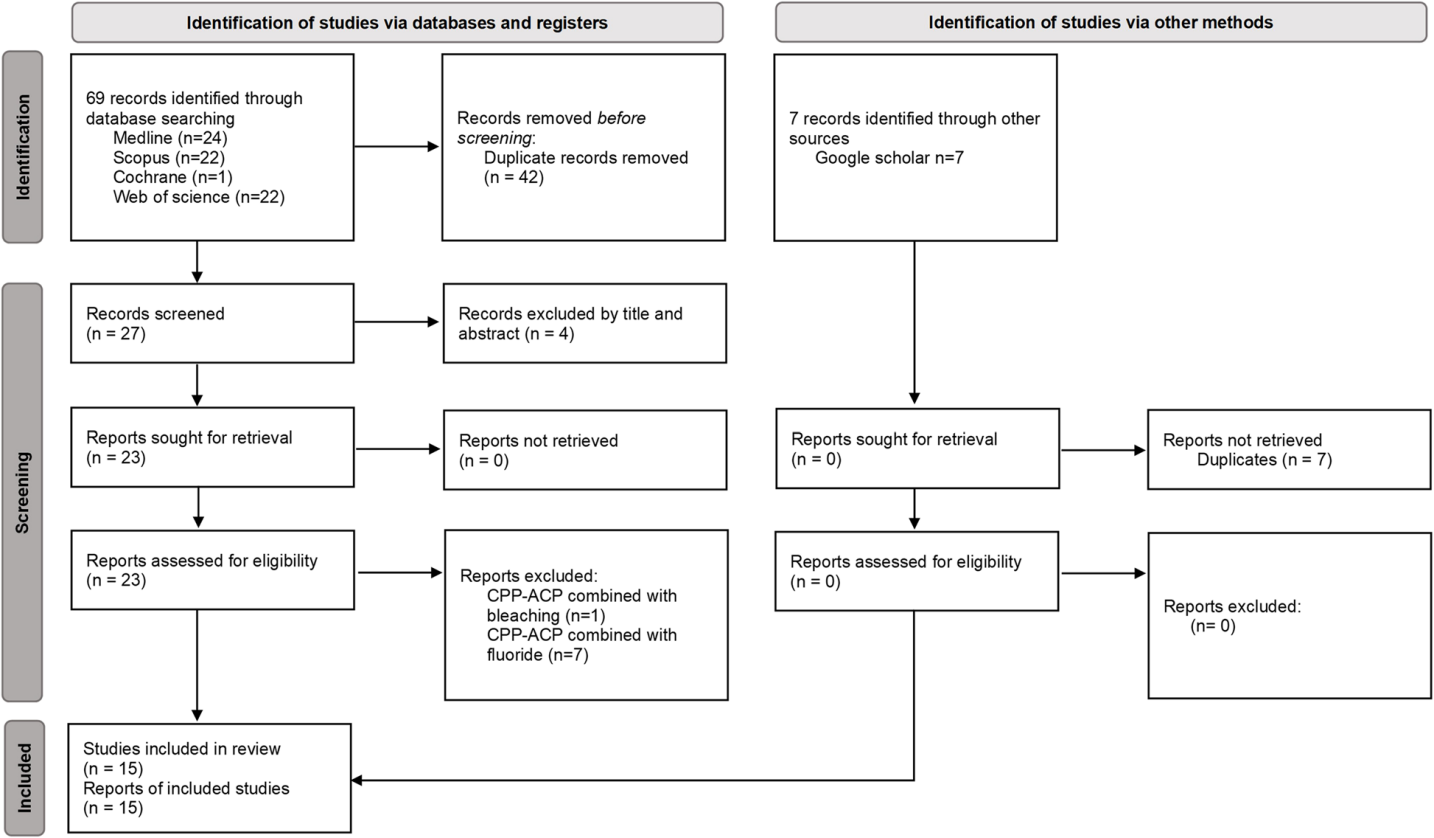
Flowchart showing the study selection process based on the PRISMA statement

The process of study selection is shown in Fig. 1. Screening the databases using the search strategy identified 76 publications: 24 from MEDLINE, 22 from Scopus, 1 from Cochrane library, 22 from Web of Science and 7 from grey literature. After duplicates exclusion 27 articles remained. Four articles were excluded based on their title and abstract. The full texts of the 23 potentially eligible articles were analysed, out of which, 8 full text articles were excluded because CPP-ACP was combined with bleaching[24] or combined with fluoride [25–31]. A total of 15 papers were deemed eligible for the systematic review[32–46].

### Study characteristics

The characteristics of the 15 studies included in the systematic review are summarized in table 2. Of the 15 studies, 6 studies evaluated the effect of CPP-ACP on the SBS when used as a preventive measure on sound enamel before bonding brackets [40–45]. In addition, 8 studies evaluated the effect of CPP-ACP on the SBS of brackets when used as a remineralizing agent on demineralized enamel [32, 33, 35–39, 46]. One study reported the SBS of brackets after using CPP-ACP for both prevention and remineralization of WSLs [34]. The SBS of 365 tooth specimens in the test groups (treated with CPP-ACP) was compared to the SBS of 1014 tooth specimens in the control groups (No pre-treatment or treatment with a different material). Separate teeth were used as controls, and none of the studies used different surfaces of the same tooth as both test and control. Twelve studies used CPP-ACP in the form of a paste [32–36, 38–41, 43, 44, 46], while the remaining three studies used a solution of CPP-ACP diluted using artificial saliva [42, 45] or deionized water [37].

**Table 2:** Summary of the characteristics of the 15 studies included in the systematic review

**Authors names**	**Year**	**Total sample size**	**Groups**	**Test group sample size**	**Enamel condition in test group**	**CPP- ACP protocol of application**	**Duration of application**	**Number of applications**	**SBS (MPa)**		**Control group sample size**	**Enamel condition in control group**	**Control groups protocol**	**SBS (MPa)**	
									**Mean**	**SD**				**Mean**	**SD**
﻿Xiaojun et al .	2009	72	4	18	Sound	Solution (Tooth Mousse+ artificial saliva, 1:10) before phosphoric acid etching + light cure adhesive (Blugloo)	60 mins	5	20.89	4.93	18	Sound	No pretreatment + Artificial saliva + phosphoric acid etching + light cure adhesive (Blugloo)	17.12	5.57
				18	Sound	Solution (Tooth Mousse+ artificial saliva, 1:10) before phosphoric acid etching + chemical cure adhesive (Unite Bonding Adhesive)	60 mins	5	27.98	9.16	18	Sound	No pretreatment + Artificial saliva + phosphoric acid etching + chemical cured adhesive (Unite Bonding Adhesive)	26.38	7.58
Tabrizi and Cakirer	2011	80	4	20	Sound	Paste (GC Tooth Mousse) before phosphoric acid etching + light cure adhesive (Light Bond)	3 mins	NR	22.57	4.32	20	Sound	No pretreatment +phosphoric acid etching + light cure adhesive (Light Bond)	21.02	5.24
											20	Sound	5% NaF varnish (Duraphat) +phosphoric acid etching + light cure adhesive (Light Bond)	14.02	4.64
											20	Sound	5% NaF varnish (Duraphat) + CPP-ACP (GC Tooth Moose) + phosphoric acid etching + light cure adhesive (Light Bond)	21.69	3.57
﻿Uysal et al	2011	80	4	20	Demineralized	Paste (GC Tooth Mousse) before phosphoric acid etching + light cure adhesive (Transbond XT)	5 mins	10	22.0	3.6	20	Sound	No pretreatment + phosphoric acid etching + light cure adhesive (Transbond XT)	24.1	4.0
											20	Demineralized	No pretreatment + Artificial saliva + phosphoric acid etching + light cure adhesive (Transbond XT)	6.6	3.9
											20	Demineralized	Flouride gel (Fluoridin N5) + phosphoric acid etching + light cure adhesive (Transbond XT)	17.1	2.9
﻿Baysal andUysal	2012	100	5	20	Demineralized	Paste (GC Tooth Mousse) before phosphoric acid etching + light cure adhesive (Transbond XT)	5 mins	10	22.0	3.6	20	Sound	No pretreatment + Phosphoric acid etching + light cure adhesive (Transbond XT)	24.1	4.1
											20	Demineralized	No pretreatment + Phosphoric acid etching + light cure adhesive (Transbond XT)	6.6	3.9
											20	Demineralized	Microabrasion + phosphoric acid etching + light cure adhesive (Transbond XT)	16.2	1.5
											20	Demineralized	Microabrasion + CPP-ACP Gel (GC Tooth Mousse) + phosphoric acid etching + light cure adhesive (Transbond XT)	24.3	1.9
Çehreli et al	2012	66	6	10	Sound	Paste (MI Paste) before phosphoric acid etching + light cure adhesive (Transbond XT)	NR	NR	5.74	1.67	10	Sound	No pretreatment+ phosphoric acid etching + light cure adhesive (Transbond XT)	8.88	1.61
				10	Sound	Paste (MI Paste) before self etching adhesive (Transbond Plus)	NR	NR	7.33	2.2	10	Sound	No pretreatment + self etching adhesive (Transbond Plus)	9.08	1.45
											10	Sound	CPP-ACPF (MI Paste plus) + phosphoric acid etching + light cure adhesive (Transbond XT)	8.82	1.54
											10	Sound	CPP-ACPF (MI Paste plus) + self etching adhesive (Transbond Plus)	8.11	1.59
Park et al	2013	60	4	15	Sound	Paste (GC Tooth Mousse) before phosphoric acid etching + light cure adhesive (Transbond XT)	3 mins	28	18.48	2.19	15	Sound	No pretreatment+ phosphoric acid etching + light cure adhesive (Transbond XT)	18.66	2.31
					Sound	Paste (GC Tooth Mousse) before self etching primer (Transbond Plus) + light cure adhesive (Transbond XT)	3 mins	28	15.51	1.71	15	Sound	No pretreatment + self etching primer (Transbond Plus) + light cure adhesive (Transbond XT)	15.75	1.77
Al-Kawari and Al-Jobair	2014	112	7	16	Sound	Paste ( MI Paste ) before phosphoric acid etching + light cure adhesive (Transbond XT)	33 mins	1	13.37	4.79	16	Sound	No pretreatment + phosphoric acid etching + light cure adhesive (Transbond XT)	11.25	4.27
				16	Sound	Paste ( MI Paste ) after phosphoric acid etching + light cure adhesive (Transbond XT)	33 mins	1	15.65	5.87	16	Sound	CPP-ACPF (MI paste plus) before phosphoric acid etching + light cure adhesive (Transbond XT)	11.05	4.85
											16	Sound	CPP-ACPF (MI paste plus) after phosphoric acid etching + light cure adhesive (Transbond XT)	16.35	3.81
											16	Sound	5% NaF varnish (Fluoraphat) before phosphoric acid etching + light cure adhesive (Transbond XT)	8.86	4.35
											16	Sound	5% NaF varnish (Fluoraphat) after phosphoric acid etching + light cure adhesive (Transbond XT)	12.56	3.74
Ladhe et al	2014	120	6	20	Sound	Solution (GC Tooth Mousse+ artificial saliva, 1:10) before phosphoric acid etching + light cure adhesive (Transbond XT)	60 mins	5	9.76	3.33	20	Sound	No pretreatment + phosphoric acid etching + light cure adhesive (Transbond XT)	10.67	4.6
				20	Sound	Solution (GC Tooth Mousse+ artificial saliva, 1:10) before phosphoric acid etching + chemical cured adhesive (Unite Bonding Adhesive)	60 mins	5	7.52	1.51	20	Sound	No pretreatment + phosphoric acid etching + chemical cured adhesive (Unite Bonding Adhesive)	10.12	4.04
											20	Sound	CPP-ACPF (GC Tooth Mousse Plus) + phosphoric acid etching + light cure adhesive (Transbond XT)	12.07	2.96
											20	Sound	CPP-ACPF (GC Tooth Mousse Plus) + phosphoric acid etching + chemical cured adhesive (Unite Bonding Adhesive)	7.36	2.54
Baka et al	2016	140	7	20	Demineralized	Paste (GC Tooth Mousse) before self etching primer (Transbond Plus)+ light cure adhesive (Transbond XT)	5 mins	10	9.04	2.64	20	Sound	No pretreatment + Self etching primer (Transbond Plus)+ light cure adhesive (Transbond XT)	10.21	2.26
											20	Demineralized	No pretreatment+ Self etching primer (Transbond Plus)+ light cure adhesive (Transbond XT)	2.26	1.46
											20	Demineralized	Fluoride gel (Bifluorid 12) + self etching primer (Transbond Plus)+ light cure adhesive (Transbond XT)	7.92	2.12
											20	Demineralized	Microabrasion (Cuxhaven) + self etching primer (Transbond Plus)+ light cure adhesive (Transbond XT)	6.18	1.65
											20	Demineralized	Microabrasion (Opalstrue) + self etching primer (Transbond Plus)+ light cure adhesive (Transbond XT)	6.54	1.83
											20	Demineralized	Resin infilteration (Icon) + self etching primer (Transbond Plus)+ light cure adhesive (Transbond XT)	10.06	2.08
﻿Velİ et al	2016	140	7	20	Demineralized	Paste (GC Tooth Mousse) before phosphoric acid etching + light cure adhesive (Transbond XT)	5 mins	10	16.2	1.4		Sound	No pretreatment + phosphoric acid etching + light cure adhesive (Transbond XT)	18.8	2
												Demineralized	No pretreatment + phosphoric acid etching + light cure adhesive (Transbond XT)	6.8	1.1
												Demineralized	Fluoride varnish (Bifluoride 12) + phosphoric acid etching + light cure adhesive (Transbond XT)	11.5	1.2
												Demineralized	Microabrasion (prepared mixture) + phosphoric acid etching + light cure adhesive (Transbond XT)	12.6	1.5
												Demineralized	Microabrasion (Opalustre) + phosphoric acid etching + light cure adhesive (Transbond XT)	14.8	1.1
												Demineralized	Resin infilteration (Icon) + phosphoric acid etching + light cure adhesive (Transbond XT)	19.1	1.4
Farhadian et al	2017	80	5	16	Demineralized	Paste (GC Tooth Mousse) before phosphoric acid etching + light cure adhesive (Transbond XT)	7 mins	20	12.53	7.16	16	Demineralized	No pretreatment + phosphoric acid etching + light cure adhesive (Transbond XT)	9.53	6.0
											16	Demineralized	CO_2_ laser irradiation + phosphoric acid etching + light cure adhesive (Transbond XT)	20.62	8.64
											16	Demineralized	CO_2_ laser irradiation before CPP-ACP (GC Tooth Mousse) + phosphoric acid etching + light cure adhesive (Transbond XT)	9.04	4.46
											16	Demineralized	CO_2_ laser irradiation through CPP-ACP (GC Tooth Mousse) + phosphoric acid etching + light cure adhesive (Transbond XT)	9.96	4.54
Gulec and Goymen	2019	80	4	20	Demineralized	Paste (GC Tooth Mousse) before phosphoric acid etching + light cure adhesive (Transbond XT)	5 mins	28	4.8	1.97	20	Sound	No pretreatment + Phosphoric acid etching + light cure adhesive (Transbond XT)	16.83	4.75
											20	Demineralized	No pretreatment + Phosphoric acid etching + light cure adhesive (Transbond XT)	13.07	3.73
											20	Demineralized	Resin infilteration (Icon) + phosphoric acid etching + light cure adhesive (Transbond XT)	4.36	2.24
Topsakal and Amuk	2019	150	10	15	Demineralized	Paste (GC Tooth Mousse) after phosphoric acid etching + light cure adhesive (Transbond XT)	3 min	NR	18.35	5.87	15	Sound	No pretreatment + phosphoric acid etching + light cure adhesive (Transbond XT)	17.96	5.26
				15	Demineralized	Paste (GC Tooth Mousse) after phosphoric acid etching + resin-modified GIC (Fuji Ortho LC)	3 min	NR	11.74	4.94	15	Sound	No pretreatment + resin-modified GIC (Fuji Ortho LC)	10.86	5.42
											15	Demineralized	No pretreatment + phosphoric acid etching + light cure adhesive (Transbond XT)	16.37	6.78
											15	Demineralized	No pretreatment + resin-modified GIC (Fuji Ortho LC)	10.87	6.88
											15	Demineralized	5% NaF varnish (Duraphat) + phosphoric acid etching + light cure adhesive (Transbond XT)	22.99	5.16
											15	Demineralized	5% NaF varnish (Duraphat) + resin-modified GIC (Fuji Ortho LC)	130.07	5.14
											15	Demineralized	Fluoride gel (Gelato APF gel) + phosphoric acid etching + light cure adhesive (Transbond XT)	15.66	5.37
											15	Demineralized	Fluoride gel (Gelato APF gel)+ resin-modified GIC (Fuji Ortho LC)	11.67	5.61
Uy et al	2019	80	10	8	Demineralized	Solution (1 gm GC Tooth Mousse + 4 ml deionized water) before phosphoric acid etching + light cure adhesive (Transbond XT) + thermocycling	24 hours	30	8.84	0.94	8	Sound	No pretreatment + Light cure adhesive (Transbond XT) + thermocycling	9.64	0.45
				8	Demineralized	Solution (1 gm GC Tooth Mousse + 4 ml deionized water) before phosphoric acid etching + light cure adhesive (Transbond XT) No thermocycling	24 hours	30	9.04	2.1	8	Demineralized	No pretreatment + Phosphoric acid etching + light cure adhesive (Transbond XT) + thermocycling	3.29	0.28
											8	Demineralized	0.21% NaF (ClinPro Tooth Crème) + phosphoric acid etching + light cure adhesive (Transbond XT) + thermocycling	8.09	1.37
											8	Demineralized	CPP ACPF (GC Tooth mousse plus) phosphoric acid etching + light cure adhesive (Transbond XT) + thermocycling	9.73	0.61
											8	Sound	No pretreatment + Phosphoric acid etching + light cure adhesive (Transbond XT) No thermocycling	11.65	1.15
											8	Demineralized	No pretreatment + Phosphoric acid etching + light cure adhesive (Transbond XT) No thermocycling	4.47	0.83
											8	Demineralized	0.21% NaF (ClinPro Tooth Crème) phosphoric acid etching + light cure adhesive (Transbond XT) No thermocycling	9.64	1.14
											8	Demineralized	CPP ACPF (GC Tooth mousse plus) phosphoric acid etching + light cure adhesive (Transbond XT) No thermocycling	11.73	1.07
﻿Daneshkazemi et al	2021	160	8	20	Sound	Paste (GC Tooth Mousse) before phosphoric acid etching + light cure adhesive (Transbond XT)	5 mins	5	13.69	4.25	20	Sound	No pretreatment + light cure adhesive (Transbond XT)	12.82	2.73
				20	Demineralized	Paste (GC Tooth Mousse) before phosphoric acid etching + light cure adhesive (Transbond XT)	5 mins	5	12.39	2.52	20	Demineralized	No pretreatment + light cure adhesive (Transbond XT)	3.51	1.37
											20	Sound	5 % NaF varnish (FlouroDose) + light cure adhesive (Transbond XT)	9.97	3.03
											20	Sound	Resin infilteration (Icon) + light cure adhesive (Transbond XT)	13.73	2.19
											20	Demineralized	5 % NaF varnish (FlouroDose) + light cure adhesive (Transbond XT)	8.34	1.66
											20	Demineralized	Resin infilteration (Icon) + light cure adhesive (Transbond XT)	12.34	4.47

*CPP-ACP *Casein phosphopeptide amorphous calcium phosphate, *CPP-ACPF *Casein phosphopeptide amorphous calcium phosphate fluoride, *MPa *Megapascals, *NaF *Sodium Fluoride, *NR *Not reported, *SBS* Shear bond strength, *SD* Standard Deviation

### Risk of bias within studies

The risk of bias assessment for each of the included studies is presented in table 3. Seven of the studies showed a low overall risk of bias [32–36, 38, 39], and 7 studies showed a medium risk [37, 40, 41, 43–46]. Only one study [42] was rated as having a high overall risk of bias. All the 15 studies [32–46] used teeth free of caries and restorations, reported randomization of the teeth to the study groups, and compared the test groups to control groups. All the studies rated as having a low risk of bias reported sample size calculation and described the procedure of sample preparation [32–36, 38, 39]. Only 7 of the studies used the CPP-ACP according to the manufacturer’s instructions [32–35, 38, 39, 43]. None of the studies reported blinding of the outcome assessor, and only one study mentioned that the bonding procedure was performed by the same investigator [36].

**Table 3:** Risk of bias assessment of the included studies

**Authors**	**Year**	**Description of sample-size calculation**	**Teeth randomization **	**Presence of a control group**	**Using sound teeth**	**Description of sample preparation**	**Using the materials according to the manufacturer’s instructions**	**Blinding of the outcome assessor**	**Bonding procedure executed by one investigator**	**Risk assesment rank**
﻿Xiaojun et al	2009	no	yes	yes	yes	yes	no	no	no	medium
Tabrizi and Cakirer	2011	no	yes	yes	yes	yes	no	no	no	medium
﻿Uysal et al	2011	yes	yes	yes	yes	yes	yes	no	no	low
﻿Baysal and Uysal	2012	yes	yes	yes	yes	yes	yes	no	no	low
Çehreli et al	2012	no	yes	yes	yes	yes	no	no	no	medium
Park et al	2013	no	yes	yes	yes	yes	yes	no	no	medium
Al-Kawari and Al-Jobair	2014	no	yes	yes	yes	yes	no	no	no	medium
Ladhe et al	2014	no	yes	yes	yes	no	no	no	no	high
Baka et al	2016	yes	yes	yes	yes	yes	yes	no	no	low
﻿Velİ et al	2016	yes	yes	yes	yes	yes	yes	no	no	low
Farhadian et al	2017	no	yes	yes	yes	yes	no	no	no	medium
Gulec and Goymen	2019	yes	yes	yes	yes	yes	yes	no	no	low
Topsakal and Amuk	2019	yes	yes	yes	yes	yes	no	no	yes	low
Uy et al	2019	no	yes	yes	yes	yes	no	no	no	medium
﻿Daneshkazemi et al	2021	yes	yes	yes	yes	yes	yes	no	no	low

### Results of individual studies and Synthesis of results

**Fig. 2:**
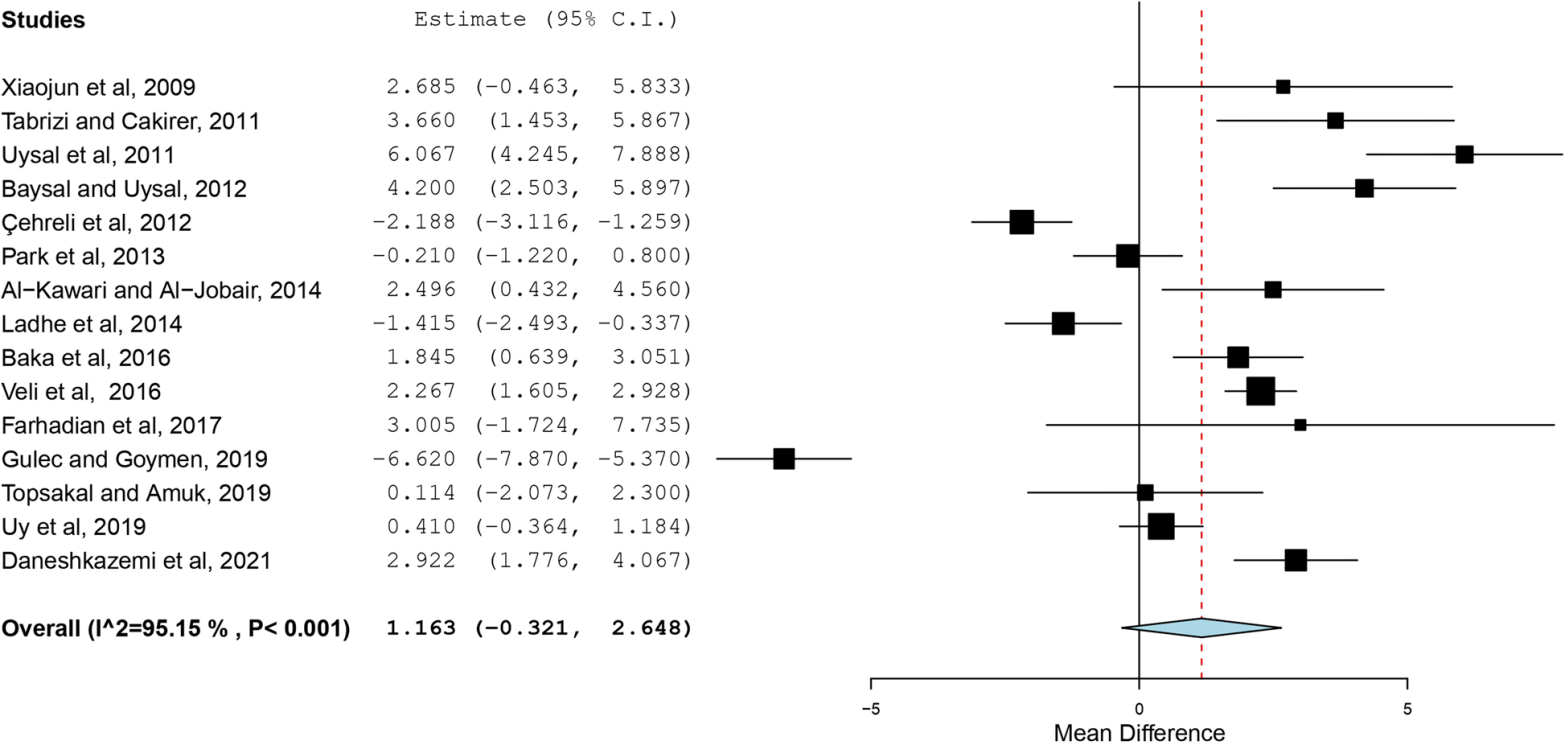
Overall forest plot comparing the reviewed studies based on standardized mean difference using a random-effects model

**Fig. 3:**
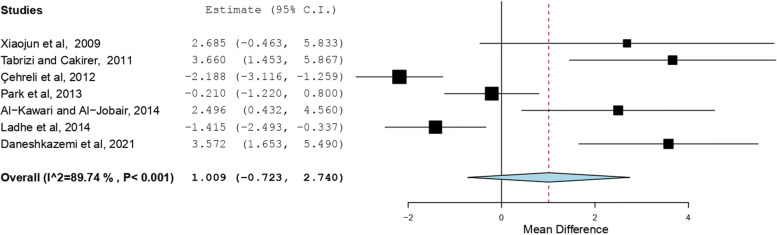
Forest plot comparing the studies using CPP-ACP for prevention of WSLs

**Fig. 4:**
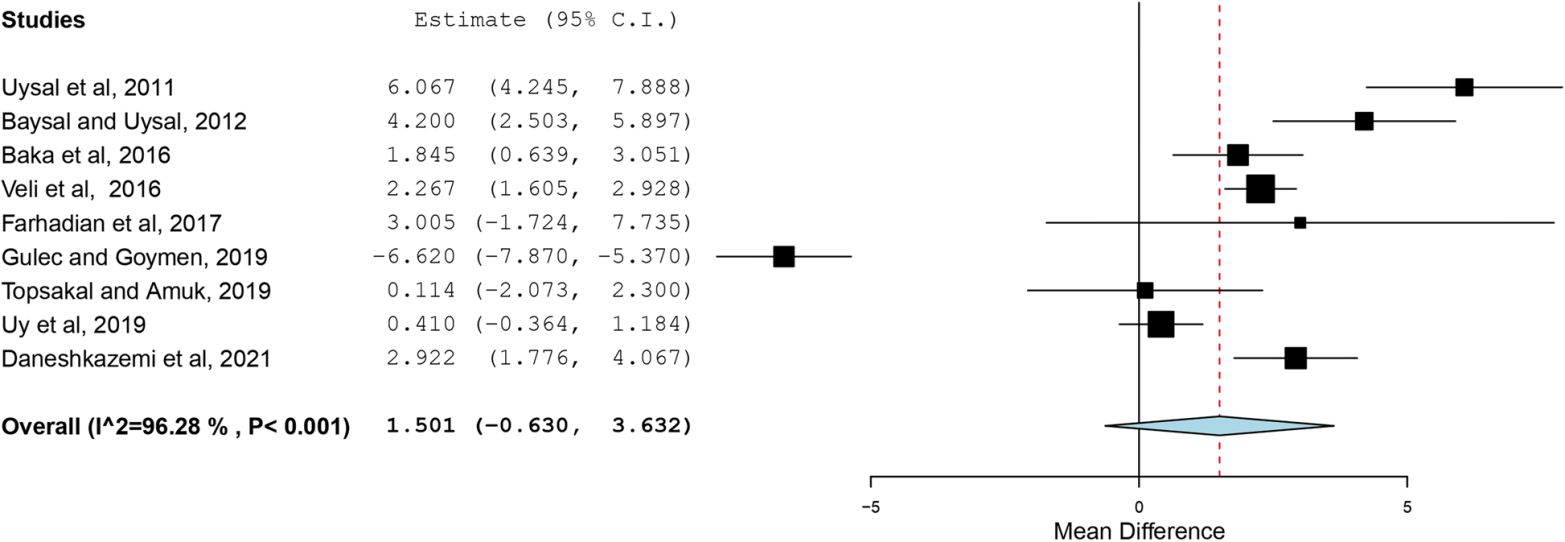
Forest plot comparing the studies using CPP-ACP for remineralization of WSLs

The SBS was the outcome evaluated in all the included studies. A summary of the findings is presented in table 2. The use of CPP-ACP prior to bonding orthodontic brackets resulted in conflicting results. The effect estimates and confidence intervals for each study are shown in Fig. 2. The overall effect of CPP-ACP on the SBS of metal orthodontic brackets was not significant with a mean difference of 1.163 MPa (SE = 0.757, 95% CI = -0.321, 2.648, *p* value = 0.125). Subgroup analysis showed that the use of CPP-ACP for prevention of WSLs before bonding did not significantly affect SBS of brackets as shown in Fig. 3 (Standardized mean difference = 1.009, SE = 0.884, 95% CI = -0.723, 2.740, *p* value = 0.254). Likewise, no significant change was found when CPP-ACP was used for remineralization of WSLs as shown in Fig. 4 (Standardized mean difference = 1.501, SE = 1.087, 95% CI = -0.630, 3.632, *p* value = 0.167).

### Risk of bias across studies

Evidence of high heterogeneity among the included studies was detected using I^2^ values and Q-Test (I^2^ = 95.147%, Q** = **288.456; df = 14; *P* < 0.001). Similarly, significant heterogeneity was observed when the use of CPP-ACP for prevention and remineralization was asssessed separately (Prevention: I^2^ = 89.736%; Q** = **58.456; df = 6; *P* < 0.001, Remineralization: I^2^ = 96.278%; Q** = **214.961; df = 8; *P* < 0.001).

## Discussion

The risk of development of WSLs during and after fixed orthodontic treatment has increased the demand for better preventive and remineralization methods. One such method is the use of CPP-ACP which can bind to the tooth surface, soft tissues and to the bacteria in the dental pellicle and plaque [15, 16]. The anti-carcinogenicity of CPP-ACP may be explained by its ability to provide a calcium reservoir creating a supersaturation state that enhances remineralization and decreases demineralization [15]. The use of CPP-ACP not only favours enamel remineralization and decrease demineralization but it may also affect the bacterial microflora [47], and result in a delay in the formation of dental biofilm [48]. The exact mechanism through which CCP-ACP affects the bacteria is uncertain but the existing evidence showed that CPP-ACP could inhibit bacterial adhesion, provide a buffering effect, and produce biofilm disruption and bacteriostatic/bactericidal effects [47].

The effect of CPP-ACP enamel pre-treatment on SBS of orthodontic brackets is debatable. Thus, this systematic review and meta-analysis was conducted to review the available literature regarding the effect of CPP-ACP enamel pre-treatment, as a preventive or remineralization method, on the SBS of metallic orthodontic brackets. Only studies performed on human enamel were included in the current review. Studies performed on bovine teeth were not included because bovine and human enamel yield significantly different SBS results due to the differences in their structural compositions[49, 50].

Screening the literature revealed 15 eligible publications [32–46]. The papers were analysed and divided according to the use of CPP-ACP into two subgroups: prevention and remineralization. Statistical analysis was conducted to compare all the eligible articles as well as each subgroup independently. Forest plots with a random-effects model were used in the current study due to the high statistical heterogeneity found among the studies.

The risk of bias of the individual studies was assessed using a modified version of the Risk of Bias tool suggested by Sarkis-Onofre et al. [22]. A recent systematic review that investigated the different tools used for quality assessment in systematic reviews of in vitro studies has highlighted the lack of a standard assessment tool [51]. The most commonly used tool was the one used in the current study [51]; however, it was modified to suit the requirements of the current investigation. Based on the risk of bias assessment, only one study was ranked as having a high risk of bias mainly due to lack of blinding of the outcome assessor, lack of description of sample size calculations or sample preparation, not using CPP-ACP according to the manufacturer’s instructions, and not reporting whether the bonding procedure was executed by one investigator. Seven of the studies were ranked as low risk as they fulfilled 6 or more parameters. All the low-risk studies described sample-size calculation, teeth randomization, and sample preparation, had a control group, and used sound teeth at the start of the study.

According to the current meta-analysis, the use of CPP-ACP, for either prevention or remineralization of WSLs, did not significantly affect the SBS of metal orthodontic brackets. Nevertheless, when CPP-ACP was used for remineralization of WSLs, the effect estimates of the majority of the included studies were positive indicating higher SBS compared to the control [32–34, 36–39, 46]. Only one study[35] was an outlier which showed a negative effect estimate. The increase in SBS when CPP-ACP was applied to demineralized enamel may be related to the ability of CPP-ACP to remineralize the subsurface lesions which increases the mineral content of the enamel, and consequently increases the bond strength(34). On the other hand, when CPP-ACP was used for prevention of WSLs the results were controversial; with four studies [34, 40, 44, 45] demonstrating positive standardized mean difference, and three studies [41–43] demonstrating negative standardized mean difference.

The SBS values reported in the 15 studies included in the current meta-analysis, following enamel pre-treatment using CPP-ACP, ranged between 4.8 MPa [35] and 27.98 MPa [45]. Clinically satisfactory orthodontic bonding has been previously reported with in vitro bond strength of 4.9 MPa [17], thus the minimum value reported by Gulec and Goymen [35], following pre-treatment of demineralized enamel using CPP-ACP, lies within the clinically accepted range of bond strength.

The wide range of reported SBS values may be explained by the inconsistency in the application protocol of CPP-ACP among the included studies. The duration of application of CPP-ACP in the form of a paste ranged between 3 min [36, 43, 44] and 33 min [40]. Other studies applied CPP-ACP in the form of a solution for 60 min [42, 45] or 30 days [37]. Several of the reviewed studies did not fully disclose the application parameters such as the duration and number of applications [36, 41, 44]. In addition, the sequence of application of CPP-ACP relative to the acid-etching procedure may have affected the SBS values. Although most of the studies included in the current review applied CPP-ACP before etching the enamel surface[32–35, 37–39, 41–46], one study applied the remineralizing agent after etching [36], and another study applied the paste before etching in one group and after etching in another group [40]. Applying CPP-ACP before performing the etching procedure may result in an enamel surface that is more resistant to acid, which may consequently affect the bonding procedure and lower the SBS values [40].

Another factor that may affect the SBS of orthodontic brackets is the type of adhesive used for bonding and the duration of photopolymerization of light-cured adhesive, where a longer polymerization time increases the SBS[18]. Moreover, the storage medium used to store the teeth during the experimental procedures may affect the results. A previous systematic review has shown that the bond strength decreased by 10.7 MPa when the samples were stored in water [18]. Thermocycling was performed as an aging process in some of the included [32, 34, 39–41] studies to evaluate the long-term bonding effectiveness. According to a previous systematic review[52], thermocycling results in a reduction in the SBS of orthodontic brackets. Another consideration is the variable speed of the crosshead of the testing machine, where a faster speed results in a lower SBS of orthodontic brackets [53]. A speed of 0.5 mm/min [33, 38, 39, 43], 1 mm/ min [32, 34–37, 40, 41, 44–46], or 3 mm/min [42] was used in the different studies.

The aforementioned disparate experimental conditions could help explain the statistically significant heterogenous results revealed during the analysis of the risk of bias across the studies.

## Limitations

One of the limitations of the current systematic review is that all the included studies were in vitro studies which do not fully simulate the conditions of the oral environment. In addition, the experimental conditions varied widely across the studies, especially the application protocol of CPP-ACP and the sequence of application of CPP-ACP relative to the acid-etching procedure. Nevertheless, it was not practical to perform separate analysis for each protocol because the number of studies analysed in the subgroups would have been inadequate. Hence, the relevance of the results of the current study to the clinical situation should be interpreted with caution. Future research should aim at mimicking the oral environment following standard guidelines to verify the results of the current systematic review and obtain clinically relevant information[18].

## Conclusions

Within the limitations of the study, the evidence suggests that the use of CPP-ACP for either prevention or remineralization of WSLs before bonding does not affect the SBS of metal orthodontic brackets.

## Supplementary Information

**Additional file 1.**

**Additional file 2.**

## Data Availability

All the data analysed during the study are included in the article.

## Abbreviations

CI: Confidence Interval
CPP-ACP: Casein phosphopeptide amorphous calcium phosphate
MPa: Megapascals
PICO: Population Intervention Comparison Outcome
PRISMA: Preferred Reporting Items for Systematic Reviews and Meta-Analyses
SBS: Shear bond strength
SD: Standard deviation
SE: Standard Error
WSL: White spot lesions
